# Exogenous spermidine affects polyamine metabolism in the mouse hypothalamus

**DOI:** 10.1515/biol-2021-0006

**Published:** 2021-01-20

**Authors:** Dongmei Jiang, Guilin Mo, Yilong Jiang, Bo Kang

**Affiliations:** College of Animal Science and Technology, Sichuan Agricultural University, Chengdu 611130, People’s Republic of China

**Keywords:** spermidine, putrescine, spermine, polyamine metabolism, hypothalamus

## Abstract

Spermidine is important for the hypothalamic control of pituitary secretion of hormones involved in neuroendocrine functions in mammals. In this study, the effect of exogenous spermidine on the expression of genes and proteins related to polyamine metabolism and polyamine levels was examined. The results indicated that treatment with spermidine at 0.05 mg/g (BW) significantly increased the levels of *Oaz*1 mRNA and protein expression and decreased putrescine content in mouse hypothalamus (*p* < 0.05). The administration with spermidine at 0.10 mg/g significantly increased the levels of *Oaz*1, *Oaz*2, and *Odc* expression in mouse hypothalamus (*p* < 0.05). Treatment with spermidine at 0.05 mg/g significantly increased the levels of *Ssat* mRNA expression and reduced the level of *Smo* mRNA expression in mouse hypothalamus (*p* < 0.05). Putrescine concentrations in the hypothalamus after the administration of spermidine at 0.10 and 0.15 mg/g were significantly higher than those in the control group (*p* < 0.05). The concentration of both spermidine and spermine in the hypothalamus after the administration of spermidine at 0.15 mg/g was decreased significantly (*p* < 0.05). In summary, our results indicate that exogenous spermidine affects polyamine homeostasis in the mouse hypothalamus by modulating the expression of genes and proteins related to polyamine metabolism.

## 1 Introduction

The polyamines spermidine, spermine, and their precursor putrescine are organic polycations present in all eukaryotes and perform various functions in different organisms [1]. The biosynthetic and catabolic pathways of polyamines in mammals are well-understood and have been described in multiple reviews [2]. Ornithine decarboxylase (ODC) catalyzes the decarboxylation of l-ornithine to produce putrescine. Putrescine is used as a substrate by constitutive spermidine synthase (SPDS), which transfers to putrescine an aminopropyl group donated by decarboxylated *S*-adenosylmethionine, which is generated by *S*-adenosylmethionine decarboxylase (SAMDC). Similarly, spermine synthase (SPMS) transfers an aminopropyl group to spermidine. Spermine oxidase (SMO) directly catalyzes the conversion of spermine to spermidine, and both spermidine/spermine *N* ^1^-acetyltransferase (SSAT) and acetylpolyamine oxidase (APAO) catalyze the conversion of spermine into spermidine and spermidine into putrescine [3,4].

In mammals, polyamines play important roles in protein and nucleic acid synthesis and stability, cell proliferation, differentiation, apoptosis, and oxidative stress [5]. In addition to serving as the biosynthetic precursor of spermine, spermidine-derived aminobutyl modification of eIF5A and subsequent hydroxylation results in the formation of a modified amino acid known as hypusine [6]. Hypusinated eIF5A is essential for the translation of mRNAs that encode proteins containing polyproline tracts [7,8]. Therefore, spermidine is an essential determinant of normal cellular translation in eukaryotes [9]. In rats, studies have shown that polyamine levels and ODC activity were higher during development and declined after growth was interrupted [10]. Thyssen and Libertun [11] reported that spermidine level was high during the first postnatal days in the rat hypothalamus, putrescine reached the highest concentration on postnatal day 6, and *α*-difluoromethylornithine (a specific and irreversible inhibitor of ODC) decreased putrescine and spermidine levels in the hypothalamus. These results indicate that spermidine is important for the hypothalamic control of the pituitary secretion of hormones involved in reproduction in mammals [10,12]. The results of our previous studies suggested that the enzyme ornithine decarboxylase antizyme (OAZ) played an important role in reproductive function mediated by the hypothalamic–pituitary–gonadal axis in female goose [13,14]. In the brain, spermidine and spermine are particularly abundant in neurons of the hypothalamus [15]. Recent studies suggested that spermidine supplementation extended the lifespan of mice and exerted cardioprotective effects by reducing cardiac hypertrophy and preserving diastolic function in old mice [16,17]. Changes in polyamine homeostasis in the hypothalamus are gaining importance in neuroendocrine and reproductive research; therefore, understanding the role of exogenous spermidine in the regulation of polyamine metabolism in the hypothalamus is essential. In this study, different doses of exogenous spermidine were administered to adult mice by intraperitoneal injection, and the expression levels of polyamine metabolism-related genes and proteins and polyamine contents were measured using quantitative real-time PCR (qRT-PCR), western blot, and high-performance liquid chromatography (HPLC). Our results demonstrated that spermidine affected polyamine metabolism in the mouse hypothalamus by modulating the expression of genes and proteins related to polyamine metabolism.

## 2 Materials and methods

### 2.1 Animals and sample collection

Thirty two female Kunming mice (6-week-old, 30 ± 3 g) were housed in eight polypropylene cages under an air-conditioned animal room (24 ± 2°C) at a relative humidity of 45 ± 5% with a 12h light–dark cycle. These animals were fed a laboratory diet and drinking water. Two cages of mice were assigned to one specific spermidine treatment, and each group was composed of four animals. The control group (*n* = 8) and three groups (*n* = 8, each) were treated with 0.9% sodium chloride and exogenous spermidine (Sigma-Aldrich, Shanghai, China) at 0.05, 0.10, and 0.15 mg/g (body weight), respectively. Spermidine was dissolved in 0.9% sodium chloride to a volume of 300 µL and then injected intraperitoneally. Animals were euthanized under general anesthesia (diethyl ether) 24 h after spermidine administration. Hypothalamus samples were bordered by the caudal edge of the mammillary bodies, the hypothalamic fissures, the rostral edge of the optic chiasm, and extended dorsally 1–2 mm.


**Ethical approval:** The research related to animal use has been complied with all the relevant national regulations and institutional policies for the care and use of animals and has been approved by the Animal Ethics Committee of the College of Animal Science and Technology, Sichuan Agricultural University (Chengdu, China).

### 2.2 Total RNA extraction and qRT-PCR

Total RNA was isolated from hypothalamic tissues using the RNAiso Plus kit (Takara, Dalian, China) according to the manufacturer’s instructions. Reverse transcription to obtain cDNA was performed using a PrimeScript™ RT reagent kit with a gDNA Eraser (Takara). Primers used were synthesized in BGI Company (Shenzhen, China) (Table 1). The qRT-PCR was carried out using the iQ SYBR Green Supermix kit (Bio-Rad Laboratories, CA, USA) in a 96-well iCycle CFX96 (Bio-Rad Laboratories). The reaction containing 5.0 µL of SYBR®Green Supermix, 4.1 µL of RNase-free water, 0.5 µL of cDNA, and 0.2 µL of each of the primers was performed as follows: 95°C for 3 min; 40 cycles of 95°C for 10 s; 58.0–62.2°C (according to Table 1) for 30 s; and 72°C for 30 s, followed by measuring the melting curves. The expression level of each gene was normalized by *Gapdh* expression level and expressed as arbitrary unit (AU). Relative quantization of gene expression was performed in three replicates for each sample.

**Table 1:** Primer sequences for mice used in the study

Gene	Primer sequence (5′–3′)	Amplicon size (bp)	*T*_m_ (°C)
*Azin*1	F: CTTTCCACGAACCATCTGCT	96	61.4
	R: TTCCAGCATCTTGCATCTCA		
*Azin*2	F: GCTTAGAGGGAGCCAAAGTG	104	62.2
	R: CTCAGCAAGGATGTCCACAC		
*Oaz*1	F: GAGTTCGCAGAGGAGCAACT	101	60.0
	R: CCAAGAAAGCTGAAGGTTCG		
*Oaz*2	F: AGTAAGTGTCCCCAGCTCCA	87	61.4
	R: ATCTTCGACAGTGGGTGAGG		
*Odc*	F: TTGACTGCCACATCCTTG	199	58.0
	R: GCTCTGCTATCGTTACACT		
*Samdc*	F: TCATGAAGCCTTCTCACCAAGGGT	155	59.0
	R: TCGGCTCTCTGGGAAATCCAAAGT		
*Spds*	F: ACCAGCTCATGAAGACAGCACTCA	189	60.0
	R: TGCTACACAGCATGAAGCCGATCT		
*Spms*	F: TTCGGGTGACTCAGTTCCTGCTAA	199	60.0
	R: AACGGAGACCCTCCTTCAGCAAAT		
*Ssat*	F: TGCCGGTGTAGACAATGACAACCT	114	59.0
	R: TAAAGCTTTGGAATGGGTGCTCGC		
*Apao*	F: AGTCTTCACATGTGCTCTGTGGGT	131	59.0
	R: TGGCAATTGTGGGTTTCCTGTCAC		
*Smo*	F: TCTGCACAGAGATGCTTCGACAGT	129	59.0
	R: TTGAGCCCACCTGTGTGTAGGAAT		
*Gapdh*	F: AACGACCCCTTCATTGAC	191	58.0
	R: TCCACGACATACTCAGCAC		

### 2.3 Western blot

Hypothalamus tissue was lysed in RIPA buffer and protein concentration was determined by BCA assay. Equal amount of proteins (approximately 30 µg) per sample were separated using 10% SDS-PAGE, transferred electrophoretically onto the polyvinylidene membrane (Bio-Rad), and blocked with 5% nonfat dry milk in Vertical Electrophoresis Systems (Bio-Rad). The primary antibodies used in this study were OAZ1 (A744, 1:1,000 dilution; ABclonal, Wuhan, China), SSAT (10708-1-AP, 1:500 dilution; Proteintech, Wuhan, China), and β-actin (A3854, 1:50,000 dilution; Sigma-Aldrich). The membrane was incubated with the primary antibody solution overnight at 4°C and then washed with the TBST. The corresponding secondary antibody (A028, 1:1,000 dilution; Beyotime, Shanghai, China) was added and incubated at room temperature for 2 h. The protein bands were visualized by using the BeyoECL Plus (a chemiluminescence reaction; Beyotime, China) in Gel Imaging Systems with an Image Lab software (Bio-Rad, USA). The bands were quantified using an ImageJ software (NIH, USA).

### 2.4 Measurement of polyamine contents

Polyamine contents were measured by HPLC using an Agilent 1100 Series system (Agilent Technologies, CA, USA) following a benzoylation procedure as described previously [18]. A 20 µL of benzoyl polyamines were separated on a 5 μm particle size C18, 4.6 × 250 mm column (Agilent Technologies). The proportion of mobile phase A (methyl alcohol) and B (water) was 62:38. The isocratic elution was performed as follows: 17 min, 62% mobile phase A. The temperature of the column was maintained at 25°C. The flow rate was at 1 mL/min. The polyamine peaks were detected with a fluorescence detector at 229 nm. Results were compared to the internal standard (1,6-hexanediamine, cat. no. 124-09-4; Sigma-Aldrich) and the standard curves for putrescine (cat. no. 51799), spermidine (cat. no. 49761), and spermine (cat. no. 55513) standards (Sigma-Aldrich).

### 2.5 Statistical analysis

Data are presented as mean ± standard error of mean (SEM). Statistical analysis was performed by one-way analysis of variance using the SAS 9.2 statistical software for Windows (SAS Institute Inc, NC, USA) followed by Duncan’s multiple range test. A *p* < 0.05 was considered to be statistically significant.

## 3 Results

### 3.1 Effect of spermidine on polyamine biosynthesis in the mouse hypothalamus

**Figure 1:**
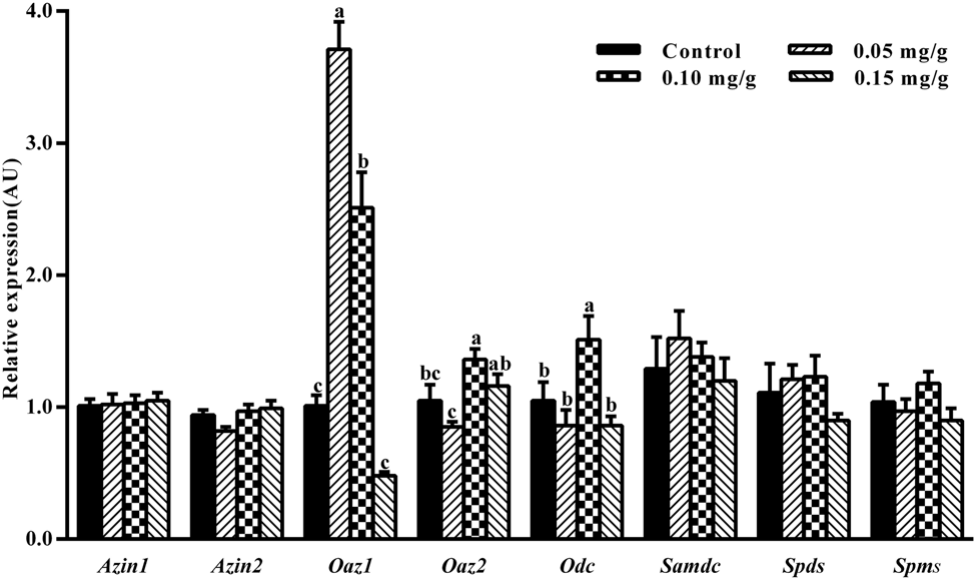
Effect of spermidine on expression levels of genes related to polyamine biosynthesis in the mouse hypothalamus. Expression levels of genes related to polyamine biosynthesis were normalized to glyceraldehyde-3-phosphate dehydrogenase (GAPDH) and presented in AU. Values are mean ± SEM. Bars with different letter are significantly different between spermidine administration and control group (*p* < 0.05).

The effect of exogenous spermidine on polyamine biosynthesis was assessed by measuring the mRNA expression levels of genes ornithine decarboxylase antizyme inhibitor 1 (AZIN1), *Azin*2, *Oaz*1, *Oaz*2, *Odc*, *Samdc*, *Spds*, and *Spms*. The mRNA expression level of *Oaz*1 in the mouse hypothalamus after the administration of spermidine at 0.05 and 0.10 mg/g was 3.71- and 2.51-fold, respectively, compared to the control group (*p* < 0.05) (Figure 1). No significant difference in the *Oaz*1 expression level was found between mice treated with 0.15 mg/g spermidine and control mice (*p* > 0.05). Furthermore, the level of OAZ1 protein in the mouse hypothalamus after the administration of spermidine at 0.05 mg/g was 1.49-fold compared to the control group (*p* < 0.05), while the level of OAZ1 protein in mice treated with 0.10 and 0.15 mg/g spermidine was not significantly different compared to the control group (*p* > 0.05) (Figure 3). As the dosage of spermidine administration increased, the level of OAZ1 protein expression showed a decreasing trend, as much as the amount of *Oaz*1 mRNA expression (Figures 1 and 3). The mRNA expression levels of *Oaz*2 and *Odc* after the administration of spermidine at 0.10 mg/g were 1.36- and 1.51-fold, respectively, compared to the control group (*p* < 0.05) (Figure 1). There was no significant difference in the mRNA expression levels of *Oaz*2 and *Odc* by treatment with spermidine at 0.05 and 0.15 mg/g compared to control mice (*p* > 0.05) (Figure 1). Moreover, there was no significant difference in the mRNA expression levels of *Azin*1, *Azin*2, *Samdc*, *Spds,* and *Spms* in the hypothalamus of mice treated with spermidine compared to control mice (*p* > 0.05) (Figure 1).

### 3.2 Effect of spermidine on polyamine catabolism in the mouse hypothalamus

**Figure 2:**
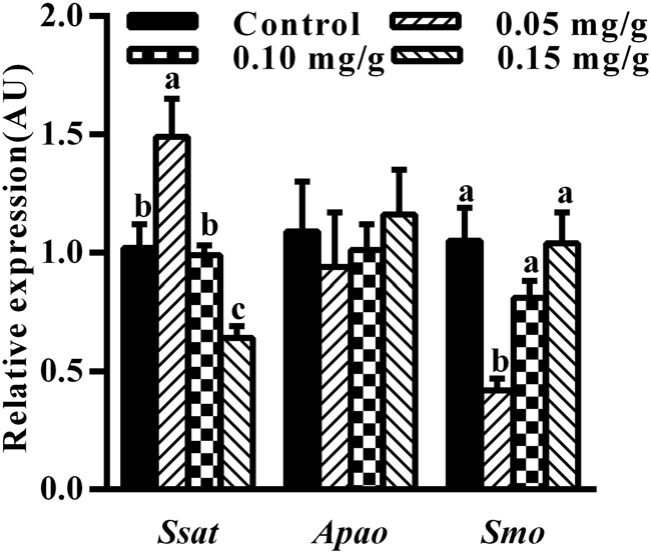
Effect of spermidine on expression levels of genes related to polyamine catabolism in the mouse hypothalamus. Expression levels of genes related to polyamine catabolism were normalized to GAPDH and presented in AU. Values are mean ± SEM. Bars with different letter are significantly different between spermidine administration and control group (*p* < 0.05).

**Figure 3:**
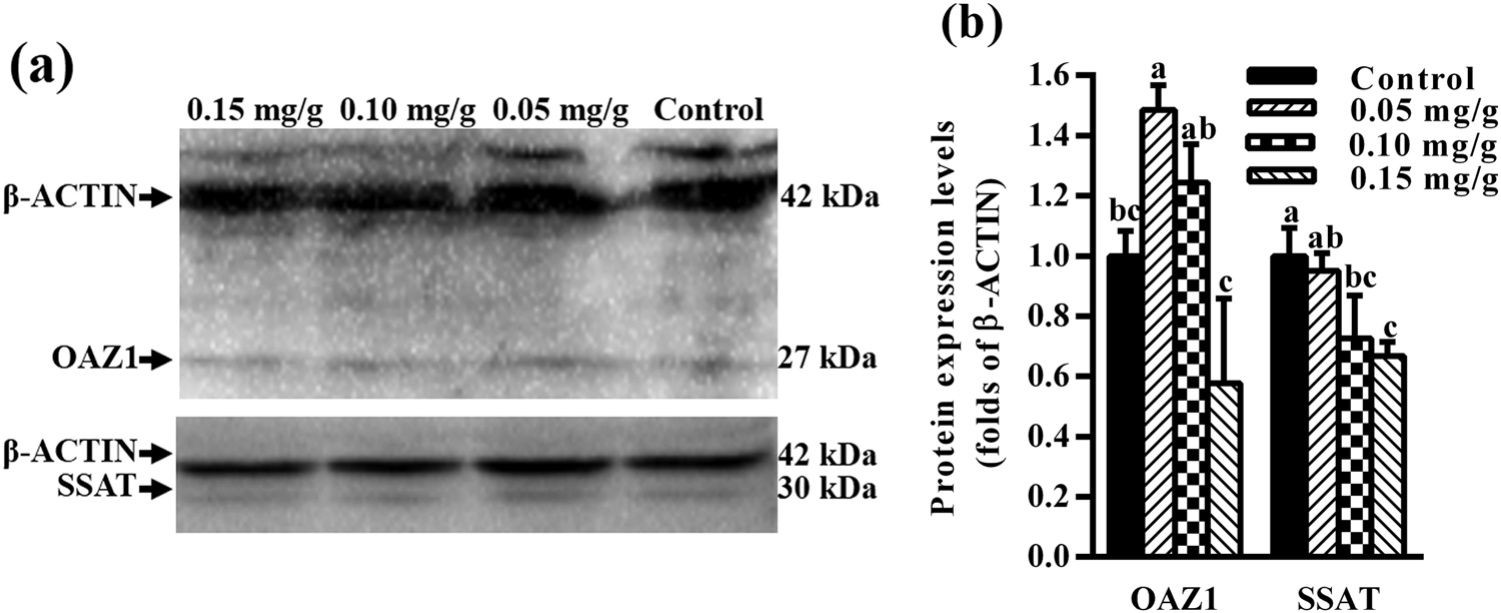
Effect of spermidine on OAZ1 and SSAT protein expression in the mouse hypothalamus. Mice were treated with spermidine at 0.05, 0.10, and 0.15 mg/g (body weight) for 24 h. (a) The expression of OAZ1, SSAT, and β-actin proteins in the hypothalamus was determined by western blot. (b) The expression of OAZ1 and SSAT proteins was quantified by densitometry, and data were normalized to β-actin. Values are mean ± SEM. Bars with different letter are significantly different (*p* < 0.05).

The mRNA expression level of *Ssat* in the hypothalamus after the administration of spermidine at 0.05 and 0.15 mg/g was 1.49- and 0.64-fold, respectively, compared to the control group (*p* < 0.05) (Figure 2). Furthermore, the level of SSAT protein in the hypothalamus after the administration of spermidine at 0.10 and 0.15 mg/g was 0.73- and 0.67-fold, respectively, compared to the control group (*p* < 0.05) (Figure 3). However, there was no significant difference in *Apao* expression level between the spermidine-treated and the control group (*p* > 0.05) (Figure 2). The mRNA expression level of *Smo* in the mouse hypothalamus after the administration of 0.05 mg/g spermidine was 0.42-fold compared to the control group (*p* < 0.05) (Figure 2).

### 3.3 Exogenous spermidine alters polyamine contents in the mouse hypothalamus

**Figure 4:**
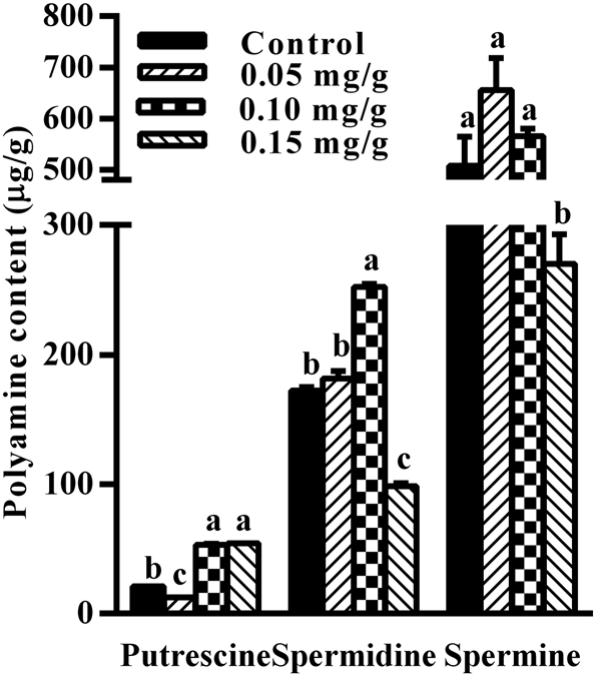
Effect of spermidine on polyamine contents in the mouse hypothalamus. Values are mean ± SEM. Bars with different letter are significantly different between spermidine administration and control group (*p* < 0.05).

Putrescine contents in the hypothalamus were significantly lower in mice treated with 0.05 mg/g spermidine compared to the control group (*p* < 0.05). It was significantly higher in mice treated with 0.10 and 0.15 mg/g spermidine compared to control mice (*p* < 0.05) (Figure 4). Spermidine contents were significantly increased in the hypothalamus after the administration of 0.10 mg/g spermidine (*p* < 0.05), but were significantly decreased after the administration of 0.15 mg/g spermidine (*p* < 0.05). Spermine content was significantly decreased in the hypothalamus after the administration of 0.15 mg/g (*p* < 0.05).

## 4 Discussion

The intracellular accumulation of polyamines can induce apoptosis and lead to toxicity in various cell types [4]. Therefore, the maintenance of polyamine homeostasis is critical in different organisms [19,20]. OAZ binds to the ODC subunit, targets ODC to ubiquitin-independent degradation by the 26 S proteasome [21,22], and inhibits polyamine transport across the plasma membrane. In our study, *Oaz*1 mRNA expression after treatment with 0.05 and 0.10 mg/g spermidine and *Oaz*2 mRNA expression after treatment with 0.10 mg/g spermidine were significantly increased. The level of OAZ1 protein after the treatment of spermidine at 0.05 mg/g was upregulated significantly (*p* < 0.05), while putrescine content decreased. Polyamines promote *Oaz* expression via the stimulation of programmed +1 ribosomal frameshifting that combines two different open reading frames to produce a full-length functional protein [23]. Our results demonstrated that exogenous spermidine at 0.05 mg/g decreases putrescine content by upregulating the expression of *Oaz*1 mRNA and protein and that the expression of *Oaz*1 mRNA and protein was more susceptive to exogenous spermidine than *Oaz*2. Ray et al. observed that the intracellular spermidine levels were sensed by the ribosomal frameshifting mechanism controlling *Oaz* translation [24]. These results suggest that spermidine supplementation can regulate the expression of *Oaz* mRNA and protein expression in mouse hypothalamus. The level of *Oaz*1 mRNA expression after 0.10 mg/g spermidine treatment increased, whereas the amount of OAZ1 protein has no significant difference. That indicates that 0.10 mg/g spermidine did not promote *Oaz*1 translation by the ribosomal frameshifting mechanism, and in turn was incapable of downregulating putrescine content in mouse hypothalamus. However, the reason why the mRNA expression level of *Oaz*1 was not increased and putrescine content increased in mice treated with 0.15 mg/g has not been determined. Except for the increased level of *Odc* in the mouse hypothalamus after the administration of 0.10 mg/g spermidine, there was no significant difference in the mRNA expression levels of *Azin*1, *Azin*2, *Odc*, *Samdc*, *Spds*, and *Spms* between the spermidine-treated and control group. The levels of *Spds* and *Spms* are not rate limiting for polyamine biosynthesis *in vivo* and are unlikely to exert significant regulatory effects on cellular polyamine levels [25]. Our results indicate that exogenous spermidine primarily regulates the expression of *Oaz*1 and *Oaz*2, particularly *Oaz*1 mRNA and protein expression, which in turn affects polyamine pool in the mouse hypothalamus.

In this study, exogenous spermidine affected *Ssat* and *Smo* mRNA expression, but did not change the mRNA expression level of *Apao* in mouse hypothalamus. SSAT acetylates spermine and spermidine to form *N* ^1^-acetylspermine and *N* ^1^-acetylspermidine, respectively. The latter can be either exported from the cell or serve as substrates for APAO [26]. SSAT is strongly regulated and highly inducible by polyamines [27,28]. Our results indicate that exogenous spermidine affects significantly the level of *Ssat* mRNA expression and protein in the mouse hypothalamus in a dose-dependent manner *in vivo* and that the SSAT levels are regulated not only at the translation and protein levels but also at the transcription level [29,30]. In addition, no significant changes in *Apao* mRNA expression in the hypothalamus of mice treated with exogenous spermidine were observed. One possible explanation is that excess polyamines are excreted by transmembrane transporter.

The permeability of a substance through the blood–brain barrier depends on its lipid solubility and the presence of a specific carrier system. Polyamine transport is highly restricted by the blood–brain barrier but is not blocked [31]. In the present study, exogenous spermidine at 0.05 mg/g increased *Oaz*1 mRNA and protein expression and decreased putrescine levels, whereas spermidine at 0.10 and 0.15 mg/g increased putrescine levels in the mouse hypothalamus. It is possible that the increased levels of OAZ1 inhibit ODC activity and then reduce the anabolic activity of putrescine. However, exogenous spermidine at a dose of 0.10 mg/g increased *Oaz*1 mRNA expression and putrescine levels, whereas the level of OAZ1 protein showed no significant difference in the mouse hypothalamus. One possible explanation for this discrepancy is that spermidine supplementation also affects polyamine pool through the putrescine transport system in the mouse hypothalamus. In addition, the levels of both spermidine and spermine in the hypothalamus were decreased after treatment with 0.15 mg/g spermidine. It indicates that exogenous spermidine affects polyamine pool in the mouse hypothalamus and may penetrate the blood–brain barrier via an undetermined pathway. A suitable experimental design needs to be established to elucidate this pathway. Polyamines are classified in the following order for their ability to enter leukemic cells and the toxicity level: spermine > spermidine > putrescine [32]. In the present study, we have shown that spermidine and spermine levels were decreased significantly after the administration of 0.15 mg/g spermidine. It can be hypothesized that the higher dose of spermidine enhanced the activity of the polyamine transport system to alleviate the toxicity of excess polyamines and thus promoted polyamine efflux. Further research is needed to elucidate this hypothesis.

In summary, our results indicated that exogenous spermidine affected polyamine homeostasis in the mouse hypothalamus by modulating the expression of genes and proteins related to polyamine metabolism. The regulation of the mechanism of polyamine transport by exogenous spermidine in the hypothalamus needs to be elucidated because of the importance of polyamines in growth and development.
